# The Application of Large Language Models in Gastroenterology: A Review of the Literature

**DOI:** 10.3390/cancers16193328

**Published:** 2024-09-28

**Authors:** Marcello Maida, Ciro Celsa, Louis H. S. Lau, Dario Ligresti, Stefano Baraldo, Daryl Ramai, Gabriele Di Maria, Marco Cannemi, Antonio Facciorusso, Calogero Cammà

**Affiliations:** 1 Department of Medicine and Surgery, University of Enna ‘Kore’, 94100 Enna, Italy; 2 Gastroenterology Unit, Umberto I Hospital, 94100 Enna, Italy; 3 Gastroenterology and Hepatology Unit, Department of Health Promotion, Mother & Child Care, Internal Medicine & Medical Specialties, University of Palermo, 90133 Palermo, Italy; ciro.celsa@unipa.it (C.C.); gabriele.dimaria@unipa.it (G.D.M.); calogero.camma@unipa.it (C.C.); 4 Department of Surgery & Cancer, Imperial College London, Hammersmith Hospital, London W12 0NN, UK; 5 Department of Medicine and Therapeutics, Faculty of Medicine, The Chinese University of Hong Kong, Hong Kong SAR, China; louishslau@cuhk.edu.hk; 6 Institute of Digestive Disease, The Chinese University of Hong Kong, Hong Kong SAR, China; 7 Digestive Endoscopy Service, Department of Diagnostic and Therapeutic Services, IRCCS—ISMETT, 90127 Palermo, Italy; dligresti@ismett.edu; 8 Department of Endoscopy, Barretos Cancer Hospital, Barretos 14784-400, Brazil; stefano.baraldo@hospitaldeamor.com.br; 9 Division of Gastroenterology, Hepatology, and Endoscopy, Brigham and Women’s Hospital, Harvard Medical School, Boston, MA 02115, USA; dramai@bwh.harvard.edu; 10 Independent Researcher, 93100 Caltanissetta, Italy; 11 Gastroenterology Unit, Department of Medical Sciences, University of Foggia, 71122 Foggia, Italy; antonio.facciorusso@virgilio.it

**Keywords:** large language models, artificial intelligence, gastroenterology, endoscopy

## Abstract

**Simple Summary:**

Large language models (LLMs) are revolutionizing the field of medicine, particularly in Gastroenterology, by improving access to information, diagnostics, treatment customization, and medical education. They analyze extensive medical data to enhance decision-making, patient outcomes, and educational tasks. While LLMs face challenges such as incomplete data, varying response accuracy, and reliance on specific input wording, they have shown promising results. However, their full integration into medical practice requires further research and regulation. Moreover, the successful integration of LLMs into medical practice necessitates customization to specific medical contexts and adherence to guidelines. This review focuses on the current evidence supporting the use of LLMs in Gastroenterology, emphasizing their potential and limitations.

**Abstract:**

Large language models (LLMs) are transforming the medical landscape by enhancing access to information, diagnostics, treatment customization, and medical education, especially in areas like Gastroenterology. LLMs utilize extensive medical data to improve decision-making, leading to better patient outcomes and personalized medicine. These models are instrumental in interpreting medical literature and synthesizing patient data, facilitating real-time knowledge for physicians and supporting educational pursuits in medicine. Despite their potential, the complete integration of LLMs in real-life remains ongoing, particularly requiring further study and regulation. This review highlights the existing evidence supporting LLMs’ use in Gastroenterology, addressing both their potential and limitations. Recent studies demonstrate LLMs’ ability to answer questions from physicians and patients accurately. Specific applications in this field, such as colonoscopy, screening for colorectal cancer, and hepatobiliary and inflammatory bowel diseases, underscore LLMs’ promise in improving the communication and understanding of complex medical scenarios. Moreover, the review discusses LLMs’ efficacy in clinical contexts, providing guideline-based recommendations and supporting decision-making processes. Despite these advancements, challenges such as data completeness, reference suitability, variability in response accuracy, dependency on input phrasing, and a lack of patient-generated questions underscore limitations in reproducibility and generalizability. The effective integration of LLMs into medical practice demands refinement tailored to specific medical contexts and guidelines. Overall, while LLMs hold significant potential in transforming medical practice, ongoing development and contextual training are essential to fully realize their benefits.

## 1. Introduction

Large language models (LLMs), such as ChatGPT (Chat Generative Pretrained Transformer, OpenAI Foundation) [1], are arising as powerful tools in the field of medicine, revolutionizing the way healthcare professionals access information, diagnose conditions, tailor treatments, and improve medical education.

These advanced artificial intelligence (AI) systems leverage vast amounts of medical data to provide evidence-based insights, promote clinical decision-making, and streamline administrative tasks.

By interpreting complex medical literature and synthesizing patient data, LLMs could enhance the accuracy and efficiency of diagnostic processes, facilitate personalized medicine, and improve patient outcomes.

Their integration into healthcare settings not only empowers physicians with real-time knowledge but also augments educational initiatives, ensuring that medical professionals remain updated with the latest advancements in the fast-evolving medical landscape.

In Gastroenterology, LLMs, especially ChatGPT, have demonstrated promising results with various applications.

These tools have the potential to educate patients by providing medical information and addressing general concerns. Furthermore, they may assist physicians in decision-making processes based on the literature data and the recommendations of guidelines. Additionally, there is growing interest in using these systems to aid scientific research. Moreover, LLMs are valuable educational tools for continuous learning and professional development, enabling healthcare providers to stay updated with the latest advancements in their field.

Nevertheless, the transition of these applications from theory to real-life is still in progress, and many aspects still require further evaluation. Furthermore, there are several concerns regarding the reliability of LLMs, the reproducibility of their outputs, data protection, and the need for regulatory systems.

This review aims to summarize the current evidence regarding the use of LLMs in Gastroenterology and to assess the drawbacks, limitations, and concerns regarding their use.

In the article, we will discuss the following topics: (1) the general aspects and characteristics of LLMs; (2) the application of LLMs in Gastroenterology for patient education; (3) the effectiveness of LLMs in assisting gastroenterologists for clinical guidance; (4) the application of LLMs in scientific research and the education of healthcare professionals; and (5) the challenges and limitations of LLMs in Gastroenterology.

## 2. Definition and Key Characteristics of LLMs

### 2.1. Fundamental Concepts

LLMs are based on deep-learning architectures that process and generate natural language. These architectures are characterized by deep learning for natural language processing and generation. The intention is to enable a model to predict each subsequent term after analyzing all preceding words. They are trained using large amounts of text data to learn about the statistical functioning of languages upon which this type of prediction is based. Most modern LLMs utilize transformer—an architectural framework introduced first by Vaswani et al., enabling long-range dependency handling within texts through self-attention techniques [2].

LLMs are characterized by wide parameterization and training on large datasets. They normally have billions of parameters, which are tunable elements within the model that can be adjusted during training to minimize the prediction error. The key parameters are represented by the learning rate (i.e., the speed of weight updates), batch size (i.e., the number of examples processed simultaneously), number of layers and hidden units (that refers to model capacity), and context window (i.e., input sequence length). This multitude of parameters enables LLMs to pick up complex language structures, allowing them to carry out tasks such as text completion, translation, summarization, and even generating human-like text [3]. The key characteristics of LLMs include the following:

- Scalability: LLMs can scale up to billions of parameters, improving performance with increased size [4];
- Pre-training and Fine-tuning: LLMs are trained on extensive text datasets and can be further fine-tuned for particular tasks or fields to enhance their effectiveness [5];
- Transfer Learning: Knowledge acquired during pre-training can be transferred to new tasks with little additional training data, hence increasing efficiency and adaptability [6].

### 2.2. Historical Development and Advancements

The development of LLMs has been a progressive journey marked by several key advancements. The journey began with the pioneering work of Hochreiter and Schmidhuber in 1997, who explored the intricacies of recurrent neural networks and long short-term memory networks [7]. These models, while innovative, were constrained by their sequential structure and struggled with capturing long-term dependencies effectively. Then, in 2014, Bahdanau and colleagues introduced the attention mechanism, a revolutionary approach that allowed models to selectively focus on relevant segments of the input sequence, greatly enhancing performance [8].

The landscape of natural language processing experienced a seismic shift in 2017 when Vaswani and his team unveiled the transformer architecture [2]. This model revolutionized natural language processing (NLP) by enabling the parallel processing of sequences, vastly improving training efficiency and effectiveness. The transformer’s self-attention mechanism empowered models to assess the importance of different words within a sequence, significantly enhancing contextual understanding. Building on this foundation, Devlin and his team introduced BERT in 2019, which stands for Bidirectional Encoder Representations from Transformers [5]. BERT marked a major advancement by employing bidirectional training, allowing models to consider both preceding and succeeding words when interpreting text. This innovation was pivotal in grasping the subtleties of language.

The transformation of NLP continued with Raffel and colleagues in 2020 through the introduction of the T5 model [6]. By unifying various NLP tasks under a text-to-text framework, they simplified the model architecture and training process, further refining the field of natural language processing.

To date, LLMs have become effective tools for comprehending and generating human language, using humongous datasets and architectures like transformers.

**Figure 1:**
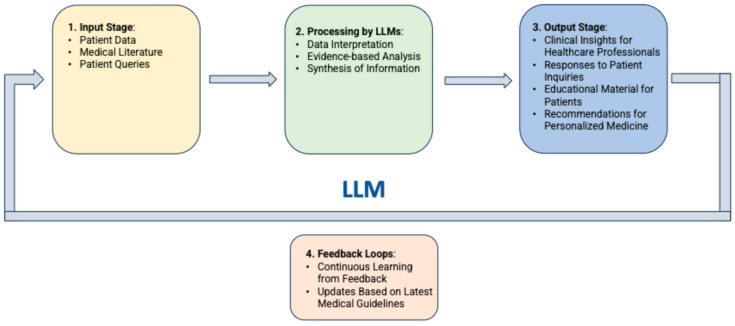
Flow diagram of LLMs operation phases for applications in medicine.

In medical applications, LLMs operate through phases including data collection and anonymization, model training with domain-specific data, input processing, inference and response generation, and integration into healthcare systems, with ongoing refinement based on feedback (Figure 1).

These models are continuously evolving over time through research that is aimed at enhancing their efficiency and scalability, as well as their applicability in different domains such as critical medical research and practice.

### 2.3. Commonly Used LLMs in Medical and Non-Medical Settings

Currently, several LLMs, crafted for medical and non-medical purposes, are available. Some of the most common are listed below.

Medical LLMs:

- MedPaLM—Developed by Google and specifically designed for medical applications.
- ClinicalBERT—An adaptation of BERT fine-tuned for clinical and biomedical texts.
- BioGPT—Microsoft’s specialized language model for the biomedical domain, designed to process and understand medical literature.
- GatorTron—Developed by the University of Florida and NVIDIA, this model is tailored for processing clinical data.

Non-Medical LLMs:

- GPT 3.5—An earlier version of OpenAI’s language models, known for its performance in natural language tasks and applications.
- GPT 4—Developed by OpenAI, this is one of the latest models in the GPT family, known for its versatility and broad applicability.
- Claude—Created by Anthropic, it is designed to be helpful, harmless, and honest for communication and interaction tasks.
- LLaMA (Large Language Model Meta AI)—Created by Meta (formerly Facebook), LLaMA focuses on efficiency and effectiveness across various general-purpose tasks.
- PaLM (Pathways Language Model)—Google’s large model designed for diverse NLP tasks, showcasing advanced capabilities in language understanding and generation.

## 3. Application of LLMs in Gastroenterology

**Figure 2:**
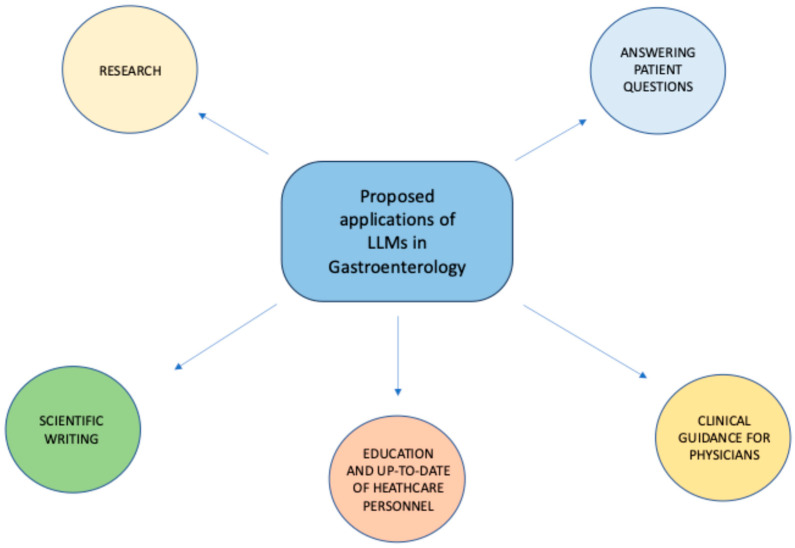
Proposed applications of LLMs in Gastroenterology.

Several studies have been conducted thus far to assess the effectiveness of LLMs in Gastroenterology across different fields, including answering patients’ common questions, assisting healthcare personnel in clinical decision-making, supporting scholars in research activities, and others (Figure 2).

To assess the available evidence, we conducted a literature search to find studies that evaluated the effectiveness of LLMs in Gastroenterology. The primary sources of the search were MEDLINE, Scopus, and the Cochrane Library, which were searched through August 2024.

### 3.1. Ability of LLMs to Answer Patients’ Questions

Recently, many researchers have assessed the accuracy of LLMs, especially ChatGPT, in responding to patients’ queries in different fields of Gastroenterology (Table 1).

#### 3.1.1. General Gastroenterology

In the general context, one study by Lahat A et al. explored the effectiveness of ChatGPT in responding to 110 common patient questions concerning Gastroenterology [9].

The study showed that ChatGPT was effective in delivering precise and comprehensive responses to patient inquiries in some instances but not in others. Regarding treatment-related questions, the average ratings for accuracy, clarity, and efficacy (on a scale of 1 to 5) were 3.9 ± 0.8, 3.9 ± 0.9, and 3.3 ± 0.9, respectively. For questions about symptoms, the mean scores for accuracy, clarity, and efficacy were 3.4 ± 0.8, 3.7 ± 0.7, and 3.2 ± 0.7, respectively. For questions concerning diagnostic tests, the mean ratings for accuracy, clarity, and efficacy were 3.7 ± 1.7, 3.7 ± 1.8, and 3.5 ± 1.7.

More recently, another study assessed the more advanced ChatGPT 4 to address 15 questions on colonoscopy and colorectal cancer (CRC) screening, 15 questions on irritable bowel syndrome (IBS), and 20 questions on inflammatory bowel disease (IBD) [10].

The study found that 84% of the answers given were generally accurate, with complete agreement among reviewers. When looking at the details, 48% of the answers were completely accurate, 42% were partially inaccurate, and 10% were accurate but with some missing information. Unsuitable references were identified for 53% of answers related to IBS, 15% of answers related to IBD, and 27% of answers related to colonoscopy and CRC prevention. In these cases, there were no suitable references for 13%, 50%, and 20% of the cases, respectively.

#### 3.1.2. Colonoscopy and Colorectal Cancer

Another area that requires implementation in the communication aspect is colonoscopy. This test is essential in the diagnosis of colon diseases and plays a key role in CRC screening. Despite this, the acceptance rate is low, mainly due to the patients’ lack of knowledge or fear of undergoing the exam.

In this regard, Lee et al. evaluated the use of ChatGPT to generate medical information in response to common patient questions on colonoscopy [11].

The authors retrieved eight questions and answers about colonoscopy from the publicly available webpages of three randomly selected US hospitals. Thereafter, they inputted these questions as prompts for ChatGPT, and the answers were rated by four Gastroenterologists for understanding, scientific adequacy, and satisfaction.

Overall, the answers were graded to be written at higher reading grade levels compared to hospital webpages. The study also found that the answers generated by AI were scientifically adequate and satisfactory overall. Moreover, ChatGPT responses had a very low text similarity (0–16%) when compared to answers found on hospital websites. However, the study was affected by the small sample size and absence of patient ratings.

Since then, LLMs have evolved, and new tools have been released, including ChatGPT 4.0.

Therefore, Tariq et al. repeated a similar study assessing LLMs’ answers to common patient inquiries on colonoscopy, comparing the performance of three LLMs (ChatGPT 3.5, ChatGPT 4, and Bard) [12].

Overall, ChatGPT 4 was considered the most effective, with 91.4% of responses graded as completely correct, 8.6% as correct but incomplete, and none of them as incorrect.

For ChatGPT 3.5 and Google’s Bard, only 6.4% and 14.9% of answers were judged as completely correct, respectively. Moreover, no responses were considered unreliable for ChatGPT 4 and ChatGPT 3.5, while two answers were considered unreliable for Bard.

This strengthens the importance of the LLM type in making these assessments, since effectiveness can vary widely from system to system and from version to version.

Another field of application of LLMs in Gastroenterology is to educate patients on the relevance of CRC screening.

For this purpose, Emile et al. posed 38 questions concerning CRC to ChatGPT [13]. The proportion of answers rated as appropriate varied among the experts: 78.9%, 81.6%, and 97.4%. Overall, at least 2 of 3 experts rated the answers as appropriate for 86.8% of questions. The agreement between raters was 79.8%. Overall, 95% of the relevant questions, 19 out of 20, that applied to the 2022 ASCRS practice parameters for colon cancer were in agreement.

A comprehensive study on the role of ChatGPT in increasing patients’ awareness to CRC screening has been recently conducted by our group [14]. In contrast to the other studies, this research involved posing 15 questions to ChatGPT 4, which were rated by 20 international experts in Gastroenterology, 20 non-experts, and 100 patients.

According to the experts’ judgment, the average accuracy score was 4.8 ± 1.1 (scale from 1 to 6). The mean completeness and comprehensibility score were 2.1 ± 0.7 and 2.8 ± 0.4 (scale from 1 to 3), respectively. Overall, the accuracy and completeness scores were significantly lower for experts compared to non-experts, while comprehensibility was similar in the two groups. Patients rated all questions as complete, comprehensible, and trustable in 97 to 100% of cases.

Concerning the comparative role of other LLMs in CRC screening, another study compared the performance of LLMs across different tools (ChatGPT, YouChat, and BingChat) [15]. The study indicated that ChatGPT and YouChat provided more reliably appropriate answers to CRC screening queries compared to BingChat.

#### 3.1.3. Pancreatic Diseases

ChatGPT was also tested in the setting of pancreatic cancer. Moazzam et al. posed 30 questions to ChatGPT regarding pancreatic cancer and its preoperative, intraoperative, and postoperative periods [16]. Each response was graded by 20 experts in hepatopancreaticobiliary surgical oncology. The majority of responses (80%) were rated as “very good” or “excellent”. Overall, 35.2% of the quality ratings were rated as “very good”, 24.5% “excellent”, and only 4.8% “poor”. Overall, 60% of experts considered ChatGPT to be a reliable information source, with only 10% indicating that ChatGPT’s answers were not comparable to those of experienced surgeons. Moreover, 90% believed that ChatGPT would replace or at least coexist with traditional search engines as the preferred source of online patient information.

#### 3.1.4. Liver Diseases

An Italian study tested ChatGPT in providing responses for patients with nonalcoholic fatty liver disease (NAFLD), more recently known as metabolic dysfunction-associated steatotic liver disease (MASLD) [17]. This is a growing global health concern and is projected to become the primary reason for liver transplant. Currently, only Resmetirom is approved as a medical treatment by regulatory agencies, but it is not available worldwide, and the main treatment recommendation remains lifestyle modification. As a consequence, there is a need for innovative methods to aid the adoption and maintenance of lifestyle changes due to the complexity of managing NAFLD. The study evaluated the ability of ChatGPT to answer 15 questions related to NAFLD, and the key opinion leaders rated the AI-generated responses.

All questions were highly comprehensible, with a mean score of 2.87 on a Likert scale of 1 to 3. Seven questions received a consistent score of 3, indicating they were easy to understand. The key opinion leaders showed a high level of agreement on the accuracy and completeness of the responses generated by the AI.

Another study aimed to assess the performance of ChatGPT in answering queries regarding cirrhosis and hepatocellular carcinoma (HCC) [18]. Two transplant hepatologists independently evaluated ChatGPT’s responses to 164 questions, with a third reviewer resolving any discrepancies.

ChatGPT demonstrated significant knowledge about cirrhosis, with a 79.1% correctness rate, and HCC, with a 74.0% correctness rate. However, only 47.3% of the responses on cirrhosis and 41.1% on HCC were deemed comprehensive. In terms of quality metrics, the model correctly answered 76.9% of the questions. However, it fell short in clearly specifying decision-making cut-offs and treatment durations. Additionally, ChatGPT demonstrated a lack of awareness about variations in guidelines, particularly related to HCC screening criteria.

Similar results were raised from another study testing ChatGPT in answering questions on liver cancer screening, surveillance, and diagnosis [19]. Overall, 48% of the answers were considered accurate, with average scores generally higher for queries related to general HCC risk factors and preventive measures. On the other side, 25% of the answers were considered inaccurate, mostly regarding LI-RADS categories. The study shows that ChatGPT often provided inaccurate information regarding the surveillance and diagnosis of HCC. It frequently gave contradictory, falsely reassuring, or outright incorrect responses to questions about specific LI-RADS categories. This could potentially influence management decisions and patient outcomes.

Liver transplantation (LT) is also a topic that arouses the interest of hepatology patients, who often inquire about their eligibility or need for LT.

Endo et al. assessed a set of 29 questions on LT that were rated by 17 abdominal transplant surgeons [20]. Overall, the majority of the 493 ratings for ChatGPT answers were categorized as “very good” (46.0%) or “excellent” (30.2%), while only a small portion (7.5%) were rated as “poor” or “fair”. Moreover, 70.6% of the experts indicated that ChatGPT’s answers were comparable to responses provided by practicing liver transplant clinicians and considered it a reliable source of information. These findings indicate that ChatGPT could serve as a valuable resource for educating patients about LT.

#### 3.1.5. Inflammatory Bowel Diseases

A recent study from Turkey assessed ChatGPT as a reliable and valuable resource for patients and healthcare professionals dealing with IBD [21].

Overall, 20 questions were created (10 concerning Crohn’s disease—CD, and 10 ulcerative colitis—UC). The questions from patients were derived from trends observed in Google searches using CD- and UC-related keywords. Questions for healthcare professionals were created by a team of four gastroenterologists. These questions focused on topics such as disease classification, diagnosis, activity level, negative prognostic indicators, and potential complications. The reliability and usefulness ratings were 4.70 ± 1.26 and 4.75 ± 1.06 for CD questions, and 4.40 ± 1.21 and 4.55 ± 1.31 for UC questions. The reliability scores given by professionals were significantly higher compared to those given by patients (*p* = 0.032).

Another common concern for patients with IBD is their diet. In this regard, Naqvi et al. posed six questions assessing key concepts regarding the dietary management of IBD to three LLMs (ChatGPT, BingChat, and YouChat) [22]. Two physicians reviewed all of the responses for appropriateness and reliability, using dietary information provided by the Crohn’s and Colitis Foundation. Overall, ChatGPT provided more reliable responses than BingChat and YouChat. While some questions received unreliable feedback from multiple LLMs, all of them advised seeking expert counsel. The agreement among raters was 88.9%.

#### 3.1.6. *Helicobacter pylori*

This is a very common topic, and patients often immediately search for information online before consulting their doctor.

In this study, Lai et al. assessed the quality of responses provided by ChatGPT for questions regarding *H. pylori* management [23]. Two reviewers assessed the responses based on categories like basic knowledge, treatment, diagnosis, and prevention. Although one reviewer scored higher than the other one in basic knowledge and treatment, there were no significant differences overall, with excellent interobserver reliability. The average score for all responses was 3.57 ± 0.13, with prevention-related questions receiving the highest score (3.75 ± 0.25). Basic knowledge and diagnosis questions scored equally at 3.5 ± 0.29. Overall, the study found that ChatGPT performed well in providing information on *H. pylori* management.

Similar results were found by another study aimed at assessing the quality of patient educational materials (PEMs) for *H. pylori* infection generated by five LLMs (Bing Copilot, Claude 3 Opus, Gemini Pro, ChatGPT 4, and ERNIE Bot 4.0) and compared with physician-sourced materials [24].

The study found that English PEMs were accurate and comprehensible but lacked completeness. Physician-sourced PEMs scored highest in accuracy, while LLM-generated English PEMs showed varied scores. Both types were similar in completeness. LLM-generated Chinese PEMs were less accurate and complete compared to English PEMs, but patients viewed them as more complete than gastroenterologists did. All PEMs were comprehensible but above the recommended sixth-grade reading level. The results of this study show that LLMs are promising resources in patient education with a need to improve completeness and adapt to different languages and contexts.

## 4. Efficacy of LLMs for Clinical Guidance

The LLMs have also been tested in more specific contexts, not concerning patient information and education, instead answering complex clinical questions based on scientific evidence and guidelines to offer clinical support to healthcare professionals (Table 2).

### 4.1. Questions from Clinicians

For instance, a recent study was conducted to test the ability of ChatGPT to respond to acute pancreatitis (AP)-related queries [25]. In particular, the study aimed to compare the agreement between ChatGPT 4.0 and ChatGPT 3.5 on objective questions related to AP. ChatGPT 4.0 had a higher accuracy rate of 94% than ChatGPT 3.5’s 80% for subjective questions and a 78.1% accuracy rate compared to ChatGPT 3.5’s 68.5% for objective questions. Overall, the agreement rate between ChatGPT 4.0 and ChatGPT 3.5 was 83.6% and 80.8%, respectively. The study highlighted the enhanced performance of ChatGPT 4.0 in answering queries related to AP compared to its earlier version.

Furthermore, the authors pointed out that when dealing with subjective questions, ChatGPT often provides a mix of relevant and irrelevant information, resulting in inconsistency and lower accuracy compared to objective questions. Nonetheless, ChatGPT 4.0 has shown improvements in offering more precise, concise, and focused answers compared to its earlier version.

In another study related to the pancreas, the performance of a customized ChatGPT for managing pancreatic cysts was evaluated in 60 clinical scenarios, which were then assessed by experts. ChatGPT agreed with the experts’ opinion in 87% of the cases. The study found no significant difference in accuracy between ChatGPT and the experts [26]. This study confirms that LLMs trained according to guidelines may represent a valuable tool for clinicians for decision-making in clinical practice.

Henson et al. assessed the performance of ChatGPT in addressing queries related to the diagnosis and management of gastroesophageal reflux disease (GERD) [27].

Of note, the responses were rated not only by gastroenterologists, but also by patients.

The study’s main findings indicate that ChatGPT offers appropriate and specific guidance for managing GERD symptoms, highlighting its potential as an interactive resource for patients and a tool for clinicians. In detail, the chatbot provided largely appropriate responses in 91.3% of cases, with the majority of responses (78.3%) including at least some specific guidance. On the other hand, patients found this tool to be useful in 100% of cases.

### 4.2. Colonoscopy Schedule

Another field of interest concerns the follow-up schedule of Gastroenterology patients, such as those undergoing screening or surveillance colonoscopy. The latter may be clear to specialists but not to non-specialists.

In this context, an initial proof-of-concept study evaluated the role of ChatGPT in enhancing post-colonoscopy management by offering recommendations based on current guidelines [28]. In this research, ChatGPT was presented with 20 clinical scenarios related to colonoscopy and pathology results. The aim was to obtain recommendations and provide simple, non-medical explanations of the results to the patient. Out of the 20 scenarios, 90% of the responses adhered to the guidelines. In total, 85% of the responses were deemed correct by both endoscopists and aligned with the guidelines. There was a strong agreement between the two endoscopists and the reference guidelines (Fleiss’ kappa coefficient = 0.84, *p* < 0.01). In 95% of the scenarios, the free-text clinical notes yielded similar interpretations and recommendations to the structured endoscopic responses.

Similarly, Chang et al. tested and compared ChatGPT 4’s accuracy, concordance, and reliability in providing colonoscopy recommendations for CRC rescreening and surveillance with current guidelines, using data from 505 colonoscopy cases [29]. Patients’ data were anonymized and inputted into ChatGPT 4. The system provided successful follow-up recommendations in 99.2% of cases. Compared to gastroenterology practices, ChatGPT 4’s recommendations aligned more closely with the US Multisociety Task Force (USMSTF) panel at 85.7% (*p* < 0.001). Among the cases where recommendations differed between ChatGPT 4 and the USMSTF panel (14.3% of cases), 5.1% suggested screenings on later dates, while 8.7% recommended earlier appointments. The inter-rater reliability between ChatGPT 4 and the USMSTF panel was strong, with a Fleiss κ of 0.786 (95% CI, 0.734–0.838; *p* < 0.001). These preliminary findings indicate that ChatGPT 4 can accurately determine routine colonoscopy screening intervals using the direct input of clinical data.

However, the main issue in using LLMs in this context is the need for proper contextualization and training according to current guidelines.

For this purpose, Lim et al. tested and compared the standard GPT 4 versus a contextualized model trained with relevant screening guidelines in the setting of screening and surveillance intervals for colonoscopy [30]. The authors found that the GPT 4 model with contextualization was superior compared to the standard GPT 4 across all domains. It did not miss any high-risk features, with only two instances experiencing hallucinations of additional high-risk features. In the majority of cases, it provided the correct interval for colonoscopy, and almost all cases appropriately cited guidelines.

In the same line, Kresevic et al. developed a customized LLM framework to interpret hepatitis C virus (HCV) clinical guidelines [31]. They found that reformatting guidelines into a uniform format, transforming tables into text-based lists, and using prompt engineering significantly improved the LLM’s accuracy in answering HCV management questions, from 43% to 99%. The framework outperformed the GPT 4 Turbo model across different question types. Interestingly, few-shot learning did not lead to additional performance improvements. These results suggest that appropriate formatting and prompting can improve the accuracy of LLMs in interpreting clinical guidelines.

### 4.3. Inflammatory Bowel Disease—Decision-Making

In the above-mentioned study by Cankurtaran et al., questions on IBD were posed to ChatGPT to evaluate the reliability and usefulness of the tool for healthcare professionals as well [21].

Professional sources received the highest ratings for reliability and usefulness, with scores of 5.00 ± 1.21 and 5.15 ± 1.08, respectively. Moreover, the reliability scores of the responses provided by professionals were significantly greater than those given by patients (*p* = 0.032).

Still, in the IBD setting and in the context of supporting healthcare professionals, other authors tested ChatGPT for its potential as a decision-making aid for acute UC in emergency settings by assessing disease severity and hospitalization needs [32]. Specifically, the authors tested the LLM’s ability to assess the severity of acute UC and determine if a specific presentation in the emergency department (ED) requires hospitalization for further treatment. The evaluation focused on 20 cases of acute UC presentations in the ED, collected over a 2-year period. ChatGPT was tasked with assessing disease severity based on the Truelove and Witts criteria for each case. The AI assessments matched 80% with expert gastroenterologists’ evaluations in 20 cases (correlation coefficient for absolute agreement = 0.839). These data suggest that ChatGPT could be a reliable support tool in clinical decision-making for UC and also in challenging clinical scenarios.

## 5. Other Applications of LLMs in Gastroenterology

### 5.1. Research Questions

One of the most interesting applications of LLMs in Gastroenterology, outside the educational and clinical areas, is research.

A study by Lahat et al. assessed the potential of ChatGPT in formulating research questions in the field of gastroenterology [33]. ChatGPT was queried on four topics, including IBD, microbiome, AI in Gastroenterology, and advanced endoscopy. The researchers found that ChatGPT was able to formulate pertinent and clear research questions, but these were not considered original. The study suggests that while LLMs can help to identify research priorities, more efforts are needed to improve the originality of the questions produced.

### 5.2. Scientific Writing

LLMs are often also used to assist researchers in writing scientific papers or research grant proposals. A survey conducted by Nature in 2023 involving 1600 researchers revealed that over 25% utilize AI to assist in writing manuscripts, and over 15% use the technology to aid in writing grant proposals [34].

No studies have been conducted thus far in this context in the field of Gastroenterology.

Nevertheless, the application of LLMs for this purpose has already raised several concerns, including the risk of inaccuracy, lack of originality, unintentional plagiarism, etc. [35]. Moreover, they may only assist in writing, not in generating substance, and some authors suggest that separating this task is not natural since writing and thinking are not separate activities [36].

### 5.3. Medical Education

Finally, LLMs could have a potential role in the education of future healthcare professionals, even if these applications remain an open research field.

In this regard, a recent Italian study evaluated ChatGPT 3.5 and Perplexity AI’s ability to answer queries from the 2023 Italian national gastroenterology residency entrance exam [37]. ChatGPT 3.5 consistently outperformed Perplexity AI, reaching a greater final score and demonstrating a strong ability to correctly answer complex questions. Both chatbots showed promise as supplementary educational tools, but their performance varied, highlighting the need for cautious use in complex scenarios. This research emphasizes the importance of ongoing investigation and the development of guidelines for integrating chatbots into medical education.

Certainly, there is currently an under-representation of LLMs’ role in research and education in Gastroenterology, and further study on this topic is necessary for the future.

## 6. Current Challenges and Future Perspectives

Despite the good performance of ChatGPT in their respective domains, these studies have some limitations that need to be discussed.

First, the results of the respective studies were limited to the tested models, input queries, and the raters’ subjective evaluation. Therefore, they cannot be generalized to the entire field or pathology, nor to the performances of different LLMs.

Secondly, it is essential to note that the responses generated by LLMs vary depending on the given prompt, leading to limited reproducibility. As a consequence, a question written in one way may generate an accurate answer compared to a slightly different question written in another way. This may affect the objective assessment of the tools. Moreover, in most studies, the questions were generated by physicians not patients. This is relevant since prompts generated by patients may be less accurate, affecting the quality of the LLM responses.

Thirdly, most of the studies were designed to evaluate the effectiveness of LLMs in responding to patients, but most of them did not include an assessment of the patients themselves. This is difficult to achieve because patients lack the skills necessary to rate the correctness and accuracy of the answers properly. They can only provide feedback on the comprehensibility, completeness, and trustworthiness of the responses.

Fourthly, not all studies indicated the language in which the interviews with ChatGPT and raters were conducted. Translating the questions into the language used by ChatGPT or translating the answers into the raters’ native language could result in evaluation differences.

Last but not least, one of the most concerning issues for the real-world application of LLMs in medicine is the ethical consideration. A major concern is patient privacy, as LLMs could potentially access sensitive patient data, risking unauthorized usage or breaches of confidentiality.

However, many organizations are releasing “open weights” versions of LLMs, in which the model’s weights and biases are publicly accessible. This enables users with adequate computing resources to download and use these models on their own systems. This has the potential to improve data protection and privacy, addressing associated concerns [38]. 

Additionally, biases inherent in the training data could lead to skewed or inequitable recommendations, disproportionately affecting underserved communities. 

The transparency of LLM decision-making processes is another critical issue, as these models often operate as “black boxes”, making it difficult for healthcare providers to understand or trust their outputs fully. Lastly, there is a possible risk of over-reliance on AI, which could diminish the role of human judgment and expertise in clinical settings, potentially leading to adverse patient outcomes if not managed correctly. Addressing these ethical concerns is essential to harness the benefits of LLMs in medicine responsibly.

Areas for improvement include ensuring model accuracy and bias reduction, particularly when dealing with diverse patient populations, and developing systems that can be run locally and provide full transparency about their development.

Finally, regulation and guidance are necessary. To date, there are no guidelines regarding the application of LLMs in gastroenterology and their routine use is not recommendable outside research settings.

## 7. Conclusions

LLMs, like ChatGPT, have the potential to transform the field of Gastroenterology by improving patient education, assisting in clinical decision-making, and advancing medical research and education. However, there are challenges in integrating LLMs into medical practice. While they can provide accurate answers based on current guidelines, the quality of their responses may vary, and biases in the training data can exist. Relying too heavily on AI poses risks, and there are ethical concerns regarding patient privacy and the transparency of decision-making. It is important to recognize these limitations to ensure that LLMs enhance human expertise rather than replace it, providing reliable support while upholding patient care standards. Ongoing research and regulation are crucial to address these challenges and effectively harness the potential of LLMs in the field of medicine.

With the proper oversight, LLMs can complement traditional methods and improve healthcare pathways in Gastroenterology, aiming to offer a reliable, real-time, and comprehensive option to engage patients and medical professionals.

**Key Issues**

- Large language models (LLMs) are emerging as powerful tools in the field of medicine, including Gastroenterology.
- Several studies have been conducted to evaluate the accuracy of ChatGPT in responding to patients’ questions about specific areas of Gastroenterology, demonstrating a good performance.
- Some studies have also been carried out to assess the role of ChatGPT in assisting physicians in clinical decision-making, showing promising results.
- Other areas of interest include the use of LLMs in research, scientific writing, and medical education in Gastroenterology, which are currently under investigation.
- Current concerns about the use of LLMs include their reliability, reproducibility of outputs, data protection, ethical issues, and the need for regulatory systems.
- To date, there are no guidelines regarding the application of LLMs in Gastroenterology, and their routine use is not recommendable outside research settings.

## Figures and Tables

**Table 1:** Overview of studies assessing the ability of LLMs to answer patients’ questions.

Author	Year	Tool	Setting	Objectives	Main Findings
Lahat [9]	2023	ChatGPT (Version not reported)	Patients’ common gastrointestinal health-related questions	To evaluate the performance of ChatGPT in answering patients’ questions, we used a representative sample of 110 real-life questions.	For questions about treatments, the average accuracy, clarity, and efficacy scores (1 to 5) were 3.9 ± 0.8, 3.9 ± 0.9, and 3.3 ± 0.9, respectively. The mean accuracy, clarity, and efficacy scores of queries concerning treatment were 3.4 ± 0.8, 3.7 ± 0.7, and 3.2 ± 0.7, respectively. The mean accuracy, clarity, and efficacy scores for diagnostic test queries were 3.7 ± 1.7, 3.7 ± 1.8, and 3.5 ± 1.7, respectively.
Kerbage [10]	2024	ChatGPT 4.0	Colonoscopy and CRC screening, IBS and IBD	To evaluate the accuracy of ChatGPT 4 in addressing frequently asked questions by patients on irritable bowel syndrome (IBS), inflammatory bowel disease (IBD), and colonoscopy and colorectal cancer (CRC) screening.	The study found that 84% of the answers given were generally accurate, with complete agreement among reviewers. When looking at the details, 48% of the answers were completely accurate, 42% were partially inaccurate, and 10% were accurate but with some missing information. Unsuitable references were discovered for 53% of answers related to IBS, 15% of answers related to IBD, and 27% of answers related to colonoscopy and CRC prevention.
Lee [11]	2023	ChatGPT 3.5	Colonoscopy	To examine the quality of ChatGPT-generated answers to common questions about colonoscopy and to assess the text similarity among all answers.	The LLM answers were found to be written at higher reading grade levels compared to hospital webpages, exceeding the eighth-grade threshold recommended for readability. The study also found that the LLM answers were scientifically adequate and satisfactory overall. Moreover, ChatGPT responses had a low text similarity (0–16%) compared to answers found on hospital websites.
Tariq [12]	2024	ChatGPT 3.5 ChatGPT 4.0 Bard	Colonoscopy	To compare the performance of 3 LLMs (ChatGPT 3.5, ChatGPT 4, and Bard) in answering common patient inquiries related to colonoscopy.	Overall, ChatGPT 4 showed the best performance, with 91.4% of its answers deemed fully accurate, 8.6% as correct but partial, and none were found incorrect. In comparison, only 6.4% and 14.9% of the responses from ChatGPT 3.5 and Google’s Bard, respectively, were rated as entirely correct. While no responses from ChatGPT 4 and ChatGPT 3.5 were viewed as unreliable, two responses from Bard were considered unreliable.
Emile, S.H. [13]	2023	ChatGPT 3.5	Colorectal cancer	To evaluate ChatGPT’s appropriateness in responding to common questions related to CRC.	Thirty-eight questions regarding CRC were posed to ChatGPT, and the appropriateness of the answers was rated differently by each expert, with percentages of 78.9%, 81.6%, and 97.4%. In general, at least two out of three experts deemed the answers appropriate for 86.8% (with a 95% confidence interval of 71.9% to 95.6%) of the questions. The inter-rater reliability was calculated at 79.8% (with a 95% confidence interval of 71.3% to 86.8%). Out of the 20 questions related to the 2022 ASCRS practice parameters for colon cancer, 19 were in agreement, amounting to a concordance rate of 95%.
Maida, M. [14]	2024	ChatGPT 4.0	Colorectal cancer screening	To evaluate ChatGPT’s appropriateness in responding to common questions related to CRC screening and its diagnostic and therapeutic implications.	Fifteen queries about CRC screening were posed to ChatGPT 4, and answers were rated by 20 gastroenterology experts and 20 non-experts in terms of accuracy, completeness, and comprehensibility. Additionally, 100 patients assessed the answers based on completeness, comprehensibility, and trustworthiness. According to the expert rating, the mean accuracy score was 4.8 ± 1.1 on a scale of 1 to 6. The mean completeness score was 2.1 ± 0.7, and the mean comprehensibility score was 2.8 ± 0.4 on a scale of 1 to 3. Compared to non-experts, experts gave significantly lower scores for accuracy (4.8 ± 1.1 vs. 5.6 ± 0.7, *p* < 0.001) and completeness (2.1 ± 0.7 vs. 2.7 ± 0.4, *p* < 0.001). Comprehensibility scores were similar between expert and non-expert groups (2.7 ± 0.4 vs. 2.8 ± 0.3, *p* = 0.546). Patients rated the responses as complete, comprehensible, and trustworthy in 97% to 100% of cases.
Atarere, J. [15]	2024	ChatGPT, YouChat, BingChat (Version not reported)	Colorectal cancer	To evaluate ChatGPT, YouChat, and BingChat’s appropriateness of responses for educating the public about CRC screening.	The main results of the study show that ChatGPT and YouChat™ provided more reliably appropriate responses to questions on colorectal cancer screening compared to BingChat. There were some questions that more than one AI model provided unreliable responses to. The study also emphasized the necessity for more in-depth evaluation of AI models in the context of patient–physician communication and education regarding colorectal cancer screening.
Moazzam, Z. [16]	2023	ChatGPT (Version not reported)	Pancreatic cancer	To characterize the quality of ChatGPT’s responses to questions pertaining to pancreatic cancer and its surgical care.	Thirty questions encompassing general information about pancreatic cancer, as well as its preoperative, intraoperative, and postoperative phases, were posed to ChatGPT. Twenty hepatopancreaticobiliary surgical oncology experts evaluated each response. The majority of responses (80%) were rated as “very good” or “excellent”. Overall, 35.2% of the quality ratings were rated as “very good”, 24.5% “excellent”, and only 4.8% “poor”. Overall, 60% of experts considered ChatGPT to be a reliable information source, with only 10% indicating that ChatGPT’s answers were not comparable to those of experienced surgeons.
Pugliese [17]	2023	ChatGPT 3.5	Nonalcoholic fatty liver disease (NAFLD)	To evaluate the accuracy, completeness, and comprehensibility of ChatGPT’s responses to NAFLD-related questions.	ChatGPT’s responses to 15 questions about NAFLD were highly comprehensible, achieving an average score of 2.87 ± 0.14 on a Likert scale of 1 to 3. Seven questions received the highest score of 3, signifying they were easy to understand. The mean Kendall’s coefficient of concordance for all questions was 0.822, reflecting strong agreement among the key opinion leaders (KOLs). A nonphysician also assessed comprehensibility, rating 13 questions as 3 and finding 2 questions somewhat difficult to comprehend.
Yeo, Y.H. [18]	2023	ChatGPT 3.5	Liver cirrhosis and hepatocellular carcinoma	To assess the accuracy and reproducibility of ChatGPT in answering questions regarding liver cirrhosis and hepatocellular carcinoma.	Two transplant hepatologists independently evaluated ChatGPT’s responses to 164 questions, with a third reviewer resolving any discrepancies. ChatGPT demonstrated significant knowledge about cirrhosis, with a 79.1% correctness rate, and hepatocellular carcinoma (HCC), with a 74.0% correctness rate. However, only 47.3% of the responses on cirrhosis and 41.1% on HCC were deemed comprehensive. In terms of quality metrics, the model correctly answered 76.9% of the questions but lacked adequate specification regarding decision-making cut-offs and treatment durations. Additionally, ChatGPT demonstrated a lack of awareness about variations in guidelines, particularly related to HCC screening criteria.
Cao, J.J. [19]	2023	ChatGPT 3.5	Liver cancer surveillance and diagnosis	To evaluate the performance of ChatGPT in answering patients’ questions related to liver cancer screening, surveillance, and diagnosis.	Out of 60 answers, 29 (48%) were deemed accurate. The average scores tended to be higher for questions concerning general HCC risk factors and preventive measures. A total of 15 of 60 (25%) answers were considered inaccurate, mostly regarding LI-RADS categories. ChatGPT often provided inaccurate information regarding liver cancer surveillance and diagnosis. It frequently gave contradictory, falsely reassuring, or outright incorrect responses to questions about specific LI-RADS categories. This could potentially influence management decisions and patient outcomes.
Endo, Y. [20]	2023	ChatGPT (Version not reported)	Liver transplantation	To evaluate the performance of ChatGPT in answering patients’ questions related to liver transplantation.	Overall, most of the 493 ratings of ChatGPT answers were classified as “very good” (46.0%) or “excellent” (30.2%), while only a small portion (7.5%) were rated as “poor” or “fair”. Moreover, 70.6% of the experts indicated that ChatGPT’s answers were comparable to responses provided by practicing liver transplant clinicians and considered it a reliable source of information.
Cankurtaran, R.E. [21]	2023	ChatGPT 4.0	Inflammatory bowel diseases	To evaluate the performance of ChatGPT as a reliable and useful resource for both patients and healthcare professionals in the context of inflammatory bowel disease (IBD).	Twenty questions were created: ten pertaining to Crohn’s disease (CD) and ten concerning ulcerative colitis (UC). The questions from patients were derived from trends observed in Google searches using CD- and UC-related keywords. Questions for healthcare personnel were created by a team of four gastroenterologists. These questions focused on topics such as disease classification, diagnosis, activity level, negative prognostic indicators, and potential complications. The reliability and usefulness ratings were 4.70 ± 1.26 and 4.75 ± 1.06 for CD questions, and 4.40 ± 1.21 and 4.55 ± 1.31 for UC questions.
Naqvi, H.A. [22]	2024	ChatGPT 3.5, BingChat, YouChat	Inflammatory bowel diseases	To evaluate the role of LLMs in patient education on the dietary management of inflammatory bowel disease (IBD).	Six questions focusing on key concepts related to the dietary management of IBD were presented to three different large language models (LLMs) (ChatGPT, BingChat, and YouChat). Two physicians evaluated all responses for appropriateness and reliability using dietary information provided by the Crohn’s and Colitis Foundation. Overall, ChatGPT provided more reliable responses on the dietary management of IBD compared to BingChat and YouChat. While there were some questions where multiple LLMs gave unreliable feedback, all of them advised seeking expert counsel. The agreement among raters was 88.9%.
Lai [23]	2023	ChatGPT 3.5	*H. pylori*	To assess the potential of ChatGPT in responding to common queries related to *H. pylori*.	Two reviewers assessed the responses based on categories like basic knowledge, treatment, diagnosis, and prevention. Although one reviewer scored higher than the other one in basic knowledge and treatment, there were no significant differences overall, with excellent interobserver reliability. The average score for all responses was 3.57 ± 0.13, with prevention-related questions receiving the highest score (3.75 ± 0.25). Basic knowledge and diagnosis questions scored equally at 3.5 ± 0.29. Overall, the study found that ChatGPT performed well in providing information on *H. pylori* management.
Zeng [24]	2024	Bing Copilot, Claude 3 Opus, Gemini Pro, ChatGPT 4, and ERNIE Bot 4.0	*H. pylori*	To assess the quality of patient educational materials (PEMs) generated by LLMs and compared with physician-sourced materials.	English patient education materials (PEMs) were accurate and easy to understand, but they fell short in completeness. PEMs created by physicians had the highest accuracy scores, whereas LLM-generated English PEMs had a range of scores. Both types had similar levels of completeness. LLM-generated Chinese PEMs were less accurate and complete than the English versions, though patients perceived them as more complete than gastroenterologists did. Although all PEMs were understandable, they exceeded the recommended sixth-grade reading level. LLMs show potential in educating patients but need to enhance their completeness and adapt to various languages and contexts to be more effective.

**Table 2:** Overview of studies assessing the efficacy of LLMs for clinical guidance.

Author	Year	Tool	Setting	Objectives	Main Findings
Du [25]	2024	ChatGPT 3.5 ChatGPT 4.0	Acute pancreatitis	To evaluate and compare the capabilities of ChatGPT 3.5 and ChatGPT 4.0 in answering test questions about AP, employing both subjective and objective metrics.	ChatGPT 4.0 had a higher accuracy rate of 94% compared to ChatGPT 3.5’s 80% for subjective questions, and a 78.1% accuracy rate compared to ChatGPT 3.5’s 68.5% for objective questions. Overall, the concordance rate between ChatGPT 4.0 and ChatGPT 3.5 was 83.6% and 80.8%,respectively. The study highlighted the enhanced performance of ChatGPT 4.0 in answering queries related to AP compared to its earlier version.
Gorelik [26]	2024	ChatGPT customized version	Pancreatic cysts	To assess the effectiveness of a custom GPT in providing integrated guideline-based recommendations for managing pancreatic cysts.	60 clinical scenarios regarding pancreatic cysts management were evaluated by a custom ChatGPT and experts. ChatGPT agreed with the experts’ opinion in 87% of the cases. The study found no significant difference in accuracy between ChatGPT and the experts.
Henson, J.B. [27]	2023	ChatGPT 3.5	Gastroesophageal reflux disease (GERD)	To assess ChatGPT’s performance in responding to questions regarding GERD diagnosis and treatment.	ChatGPT provided largely appropriate responses in 91.3% of cases, with the majority of responses (78.3%) including at least some specific guidance. On the other hand, patients found this tool to be useful in 100% of cases.
Gorelik, Y. [28]	2023	ChatGPT 4.0	Screening and surveillance intervals for colonoscopy	To evaluate ChatGPT’s effectiveness in improving post-colonoscopy management by offering recommendations based on current guidelines.	Out of the 20 scenarios, 90% of the responses adhered to the guidelines. In total, 85% of the responses were deemed correct by both endoscopists and aligned with the guidelines. There was a strong agreement between the two endoscopists and the reference guidelines (Fleiss’ kappa coefficient = 0.84, *p* < 0.01). In 95% of the scenarios, the free-text clinical notes yielded similar interpretations and recommendations to the structured endoscopic responses.
Chang, P.W. [29]	2024	ChatGPT 4.0	Screening and surveillance intervals for colonoscopy	To compared the accuracy, concordance, and reliability of ChatGPT 4 colonoscopy recommendations for colorectal cancer rescreening and surveillance with contemporary guidelines and real-world gastroenterology practice.	Data from 505 colonoscopy patients were anonymized and inputted into ChatGPT 4. The system provided successful follow-up recommendations in 99.2% of cases. Compared to gastroenterology practices, ChatGPT 4’s recommendations aligned more closely with the USMSTF Panel at 85.7% (*p* < 0.001). Among the cases where recommendations differed between ChatGPT 4 and the USMSTF panel (14.3% of cases), 5.1% suggested later screenings, while 8.7% recommended earlier ones. The inter-rater reliability between ChatGPT 4 and the USMSTF panel was strong, with a Fleiss κ of 0.786 (95% CI, 0.734–0.838; *p* < 0.001).
Lim, D.Y.Z. [30]	2024	ChatGPT 4.0	Screening and surveillance intervals for colonoscopy	To evaluate a contextualized GPT model versus standard GPT with recent guidelines, to offer guidance on the suggested screening and surveillance intervals for colonoscopy.	The GPT 4 model with contextualization outperformed the standard GPT 4 across all domains. It did not miss any high-risk features, and only two cases experienced hallucinations of additional high-risk features. In the majority of cases, it provided the correct interval for colonoscopy, and almost all cases appropriately cited guidelines.
Kresevic, S. [31]	2024	ChatGPT 4.0	Hepatitis C	To evaluate the performance of a novel LLM framework integrating clinical guidelines with retrieval augmented generation (RAG), prompt engineering, and text reformatting strategies for the augmented text interpretation of HCV guidelines.	Reformatting guidelines into a consistent structure, converting tables to text-based lists, and using prompt engineering significantly improved the LLM’s accuracy in answering HCV management questions, from 43% to 99% (*p* < 0.001). The novel framework outperformed the baseline GPT 4 Turbo model across different question types, including text-based, table-based, and clinical scenarios.
Cankurtaran, R.E. [21]	2023	ChatGPT 4.0	Inflammatory bowel diseases	To evaluate the efficacy of ChatGPT as a reliable and useful resource for both patients and healthcare professionals in the context of inflammatory bowel disease (IBD).	Twenty questions were created (ten on Crohn’s disease—CD—and ten on ulcerative colitis—UC). The questions from patients were derived from trends observed in Google searches using CD- and UC-related keywords. Questions for healthcare professionals were created by a team of four gastroenterologists. Professional sources received the highest ratings for reliability and usefulness, with scores of 5.00 ± 1.21 and 5.15 ± 1.08, respectively. Moreover, the reliability scores of the responses provided by professionals were significantly greater than those given by patients (*p* = 0.032).
Levartovsky, A. [32]	2023	ChatGPT 4.0	Inflammatory bowel diseases (ulcerative colitis)	To assess the potential of ChatGPT as a decision-making aid for acute ulcerative colitis in emergency-department (ED) settings by assessing disease severity and hospitalization needs.	The evaluation focused on 20 cases of acute UC presentations in the ED, collected over a 2-year period. ChatGPT was tasked with assessing disease severity based on the Truelove and Witts criteria for each case. The AI assessments exhibited a match of 80% with expert gastroenterologists’ evaluations in 20 cases (correlation coefficient for absolute agreement = 0.839).

## Acronyms and Abbreviations

Large language models	LLMs
ChatGPT	Chat Generative Pretrained Transformer, OpenAI Foundation
Artificial intelligence	AI
Natural language processing	NLP
BERT	Bidirectional Encoder Representations from Transformers
Colorectal cancer	CRC
Inflammatory bowel disease	IBD
Irritable bowel syndrome	IBS
Nonalcoholic fatty liver disease	NAFLD
Metabolic dysfunction-associated steatotic liver disease	MASLD
Hepatocellular carcinoma	HCC
Liver transplantation	LT
Crohn’s disease	CD
Ulcerative colitis	UC
Patient educational materials	PEMs
Acute pancreatitis	AP
Gastroesophageal reflux disease	GERD
US Multisociety Task Force	USMSTF
Hepatitis C virus	HCV
Emergency department	ED
